# Imaging of sacroiliac joints in patients with acromegaly

**DOI:** 10.1038/s41598-019-48250-w

**Published:** 2019-08-12

**Authors:** Kader Ugur, Ahmet Karatas, Burak Oz, Hakan Artas, Suleyman Aydin, Suleyman Serdar Koca

**Affiliations:** 1 Department of Endocrinology and Metabolism Diseases, Firat University School of Medicine, Elazig, Turkey; 2 Department of Rheumatology, Firat University School of Medicine, Elazig, Turkey; 3 Department of Radiology, Firat University School of Medicine, Elazig, Turkey; 4 Department of Medical Biochemistry and Clinical Biochemistry, Firat Hormones Research Group, Firat University School of Medicine, Elazig, Turkey

**Keywords:** Pituitary diseases, Ankylosing spondylitis

## Abstract

Acromegaly can lead to structural alterations of joints and bones. Patients with acromegaly may, therefore, have musculoskeletal complaints. In this study, sacroiliac joints are investigated in patients with acromegaly. 33 patients with acromegaly were enrolled. Sacroiliac joints were examined by X-ray and magnetic resonance imaging (MRI). In acromegaly, sacroiliac joints were abnormal in 36% of the patients by X-ray and 12.1% by MRI. When current axial spondylarthritis (SpA) classification criteria were taken into account, 6.1% of acromegaly patients could be classified as non-radiographic axial SpA and 2% as radiographic axial SpA. Sacroiliac joints are frequently affected in acromegaly and thus this disorder mimics the features of AS and SpA. Acromegaly should be kept in mind in the differential diagnosis of AS and SpA.

## Introduction

Acromegaly is a rare disease characterized by the increased release of growth hormone (GH) and insulin-like growth factor-1 (IGF-1). Its prevalence is 2.8–13.7/100,000, affecting men more frequently than women. Fatigue, joint pain, headache, paresthesia and sweating are frequent complaints in patients with active acromegaly. The disease may cause different systemic complications, such as cardiac, respiratory, endocrine and oncological problems, and was first described in the 16th century with some skeletal changes (e.g. vertebral body deposits, prognathism, prosopoectasia, hyperostosis) in the medical literature^1^. Therefore, it is important to determine the effects on the musculoskeletal system. Different musculoskeletal involvements, including peripheral joint pain, back pain, functional impairment in joints and tendon abnormalities, develop in these patients^2,3^. For the diagnosis of acromegaly, GH levels during oral glucose tolerance test and random IGF-1 levels should be measured. Patients who have a GH level of >1 ng/mL at the 1st h during the oral glucose tolerance test and those with IGF-1 level above the normal level for age and gender are diagnosed as acromegaly^4^.

Musculoskeletal pain, a common problem in acromegaly, is associated with a decrease in the quality of life. Approximately 50–70% arthropathy occurs in patients with acromegaly, who may have low back and hip pain due to involvement of the axial system. Furthermore, radiographically, joint space narrowing, osteophytes and enthesitis can be detected^5,6^. Spondyloarthritis (SpA) comprises a group of heterogeneous inflammatory rheumatic diseases. Human leukocyte antigen (HLA)-B27 positivity, peripheral joint involvement, sacroiliitis, spondylitis, enthesitis, dactylitis, uveitis, inflammatory bowel disease and psoriatic skin lesions are found in SpA group diseases, which are subdivided into 2 categories: axial (axSpA) and peripheral (pSpA)^7,8^. AxSpA affects mainly the sacroiliac joints and the spine, and is also categorised into 2 subtypes: non-radiographic axSpA and radiographic axSpA, including ankylosing spondylitis (AS). It is named as radiographic axial SpA if sacroiliitis is shown on X-ray; if sacroiliitis is detected by MRI but the X-ray is normal; it is considered to be a non-radiographic axSpA^9^. Peripheral SpA usually affects joints of the lower extremity, the foot and the knee. Thus, SpA causes axial and peripheral complaints that include low back pain, peripheral joint pain, and heel pain (caused by enthesitis). Radiological MRI findings of sacroiliac joints have been integral part of the axSpA diagnosis. However, the specificity of imaging is lower and, several diseases may mimic changes characteristic of axSpA^10^. As a result, acromegaly may mimic the clinical and radiological features of SpA. Therefore, patients with acromegaly were examined for similarities of axSpA in our study.

## Materials and Methods

### Participants

The study was carried out by the departments of endocrinology and radiology at Firat University. Ethical committee approval was obtained from Firat University clinical research ethics committee. We conducted our study in accordance with the approved guidelines of ethical principles for medical research involving human subjects. Written informed consent was provided from all participant. 33 acromegaly patients diagnosed by the level of serum IGF-1 (insulin-like growth factor) and an increased GH level after oral glucose tolerance test (OGTT) with 75 g glucose^11^ were included.

### Disease activity of acromegaly

Acromegaly patients were considered in remission when the GH response was <1 μg/l and there was a normal IGF-1 level according to age after 75 g OGTT. Patients with high GH and IGF-1 levels were considered as active^11^. Patients with active acromegaly and in remission were included.

### Screening for SpA

Assessment of SpondyloArthritis International Society (ASAS) criteria were used in diagnosing spondyloarthritis^12^. Acromegaly patients were checked for arthritis, uveitis, dactylitis, enthesitis, psoriasis, inflammatory bowel disease and SpA family history, and HLA-B27 was also analysed. Direct radiography and sacroiliac joint MRI were used in detecting sacroiliitis. Two blinded readers (SSK and AK) independently scored the images.

Pre- and post-gadolinium T1-weighted sequences, fat-saturated T1 and T2-weighted sequences and short tau inversion recovery (STIR) sequences were performed. MRI of the sacroiliac joints was used to assess active inflammatory lesions, including bone marrow edema (BME: osteitis), capsulitis, synovitis, enthesitis, abscess and soft tissue involvement, and chronic inflammatory lesions (including sclerosis, erosions, fat deposition and bony bridges/ankyloses). The readers judged for the presence or absence of BME according to the ASAS definition^13^, which by definition places the focus on scoring only lesions considered ‘highly suggestive of axial SpA’ as being positive. In the analysis, an MRI was considered positive if readers agreed that it met the ASAS MRI criteria for defining sacroiliitis by MRI^13^.

### Statistical analysis

Data were analyzed using the *International Business Machines - Statistical Product and Service Solutions* (IBM-SPSS, version 21.0) software (IBM Corp., Armonk, NY, USA). Demographic and clinical characteristics of the groups were determined. The Chi-square test was used for categorical data. The Mann-Whitney U test was used for the comparison of continuous measurements. Continuous data were given as mean ± standard deviation. Inter-observer agreement on positive/negative MRI of the SI joints was inspected using Cohen’s kappa (κ) coefficient.

## Results

### Baseline characteristics

The mean age of the groups was 41.9 ± 9.7 years in patients with acromegaly. 64% of the acromegaly patients were female. HLA-B27 was positive in 3.1%. 23 (69.7%) had complained about chronic back pain, but none had inflammatory back pain^14^.

### X-ray examination of sacroiliac joints

**Figure 1:**
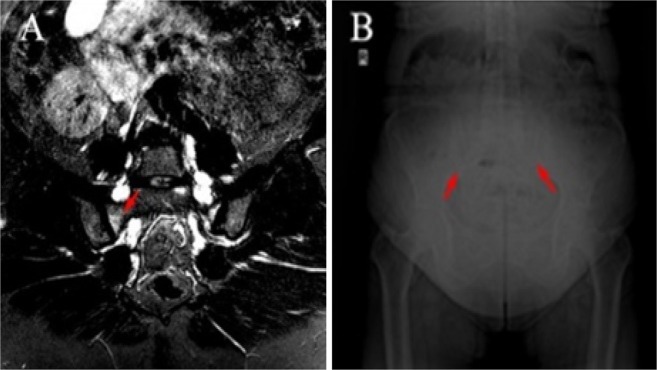
(**A**) STIR (short tau inversion recovery) sequence of MRI showing bone marrow edema in the sacroiliac joint of an acromegaly patient. (**B**) X-ray film showing sclerosis and partial ankylosis in the sacroiliac joint of an acromegaly patient.

In the X-ray graphs of sacroiliac joint, 63.6% of acromegaly patients had normal sacroiliac joints. 18.2% were evaluated as stage I, 15.2% as stage II, 3.1% as stage III sacroiliitis (Fig. 1) based on the New York sacroiliitis radiological grading criteria^15^ (Table 1). From the radiography of sacroiliac joints, one (3.1%) of the acromegaly patients met the radiological criteria of the modified New York AS criteria set^15^.

**Table 1:** Grading of radiographical findings of sacroiliitis according to New York criteria.

Grade 0	Normal
Grade 1	Suspicious changes
Grade 2	Minimal definite abnormality: small localized areas with erosion or sclerosis without change of sacroiliac joint space
Grade 3	Erosions, sclerosis, change of joint space (widening, narrowing) or partial ankylosis
Grade 4	Total ankylosis

### MRI examination of sacroiliac joints

From the MRI of sacroiliac joints, 12 (36.4%) patients had no abnormalities (Table 2). Fatty marrow deposition and sclerosis occurred 33.3 and 18.2% of the acromegaly patients, respectively. None had erosions. Active sacroiliitis was detected in 4 (12.1%) of those examined for sacroiliac MRI (Fig. 1). One was enthesitis and 3 (9.1%) were BME. BME lesions in 2 (6.1%) patients met the ASAS MRI working group’s criteria for defining sacroiliitis by MRI^13^. Thus, 3.1% of our acromegaly patients met radiographic axial SPA classification criteria, and 6.1% met non-radiographic axSpA classification criteria^14^.

**Table 2:** Radiographical and MRI findings of sacroiliac joints in patients with acromegaly.

Variables	*n* (%)
Sacroiliitis on X-ray (any grade)	12 (36.4)
- Sacroiliitis on X-ray according to New York criteria	1 (3.1)
Sacroiliitis on MRI (any abnormality)	16 (48.5)
- Sacroiliitis on MRI (bone marrow edema)	3 (9.1)
- Enthesopathy	1 (3.1)
- Sacroiliitis on MRI (erosion)	—
- Sacroiliitis on MRI (sclerosis)	6 (18.2)
- Sacroiliitis on MRI (Fatty marrow deposition)	11 (33.3)
Sacroiliitis on MRI according to ASAS definitions	2 (6.1)

MRI: magnetic resonance imaging; ASAS: assessment of spondyloarthritis international society.

Intra-observer agreements between the readers were substantial (Cohen’s κ: 0.61–0.80). The agreements were higher for the MRI evaluations of sacroiliac joints: BME (κ = 0.69, p < 0.001), sclerosis (κ = 0.76, p < 0.001), fatty marrow depositions (κ = 0.75, p < 0.001) than X-ray graph scoring of sacroiliac joints (κ = 0.62, p < 0.001).

### The disease activity of acromegaly patients

The mean disease duration of the patients was 6.5 ± 3.4 years. In terms of disease activation, 74% of them were in remission, and 26% were active. There was no significant difference between active acromegaly patients and those in remission in terms of mean age and gender distribution. There was no statistical difference between the disease activity of acromegaly and sacroiliac joints X-ray graph and MRI findings.

## Discussion

Acromegaly patients can have back pain, especially when features of pain is not diagnosed well the identification of the SpA that can accompany this group of patients and consideration of SpA in differential diagnosis seems to be the difficult part. Acromegaly is frequently recognized in advanced stage patients even with inspection. However, this typical phenotype appearance is not formed in the early stages of the disease. These patients may apply to different departments with complaints, such as joint pain, functional impairment in the joints and tendinopathy, which may lead to delays in the diagnosis of the disease. This study is important in determining whether acromegaly can mimic radiographic findings of SpA by evaluating the X-ray graphs and MRI data of the patients.

Podgosrki *et al*.^16^ found peripheral joint involvement in 74% of acromegaly patients, and spinal involvement in 47%. Acromegaly patients, who, in particular presented with joint complaints, can apply to the rheumatology departments. They may have SpA-like findings, such as back pain and Achilles tendinopathy. On the other hand, if there is an accompanying SpA table in the acromegaly patient, their complaints may be associated with acromegaly, which may delay a diagnosis of SpA.

We found a radiographic abnormality in the sacroiliac joint in 36.4% of acromegaly patients, and the MRI findings showed sacroiliitis in 48.5%. Taking the current axSpA classification criteria into account, 6.1% of acromegaly patients could be classified as non-radiographic axSpA and 3.1% as radiographic axSpA. Sacroiliitis concomitant with acromegaly has been reported^17^. There are also a small number of case reports indicating the concurrence of acromegaly and AS^18,19^. However, there are no prospective studies evaluating acromegaly patients with low back pain with sacroiliac MRI. Osteoarticular changes in acromegaly can be detected in most patients, but its pathogenesis remains unknown. GH-IGF-I complex and secondary degenerative changes cause osteoarticular changes in acromegaly patients^6^. GH and IGF-I may lead to cartilage enlargement in the cartilaginous joint and to synovial hypertrophy, followed by degenerative changes and irregularity of the sacroiliac joint, resulting in sacroiliitis-like radiographic findings.

Yoshioka *et al*.^20^ have shown that none of their acromegaly patients had HLA-B27 positivity. However, we found 3.1% of patients were HLA-B27 positive. HLA-B27 positivity in healthy controls in Turkey was 4.7–6.2%^21,22^. It seems that HLA-B27 positivity in acromegaly is similar in healthy subjects, which suggests there is no association between HLA-B27 and acromegaly.

In conclusion, this is the first study evaluating sacroiliac MRI in a large number of acromegaly cases, which is a rare disease. Sacroiliitis was detected in 6.1% of them by sacroiliac MRI. X-ray radiograph of sacroiliac joints proved abnormal in 36.4% of the patients. 69.7% of patient had chronic back pain, although none of them was of the inflammatory type. These results suggest that acromegaly can mimic SpA with clinical and radiological features in terms of both X-ray radiography and MRI scanning.
